# Near-Infrared Spectroscopy Coupled with Chemometrics and Artificial Neural Network Modeling for Prediction of Emulsion Droplet Diameters

**DOI:** 10.3390/mi13111876

**Published:** 2022-10-31

**Authors:** Filip Grgić, Tamara Jurina, Davor Valinger, Jasenka Gajdoš Kljusurić, Ana Jurinjak Tušek, Maja Benković

**Affiliations:** Faculty of Food Technology and Biotechnology, University of Zagreb, Pierottijeva ul. 6, 10000 Zagreb, Croatia

**Keywords:** microfluidic emulsification, aqueous mint extract, NIR spectra, chemometrics, ANN modeling

## Abstract

There is increased interest in the food industry for emulsions as delivery systems to preserve the stability of sensitive biocompounds with the aim of improving their bioavailability, solubility, and stability; maintaining their texture; and controlling their release. Emulsification in continuously operated microscale devices enables the production of emulsions of controllable droplet sizes and reduces the amount of emulsifier and time consumption, while NIR, as a nondestructive, noninvasive, fast, and efficient technique, represents an interesting aspect for emulsion investigation. The aim of this work was to predict the average Feret droplet diameter of oil-in-water and oil-in-aqueous mint extract emulsions prepared in a continuously operated microfluidic device with different emulsifiers (PEG 1500, PEG 6000, and PEG 20,000) based on the combination of near-infrared (NIR) spectra with chemometrics (principal component analysis (PCA) and partial least-squares (PLS) regression) and artificial neural network (ANN) modeling. PCA score plots for average preprocessed NIR spectra show the specific grouping of the samples into three groups according to the emulsifier used, while the PCA analysis of the emulsion samples with different emulsifiers showed the specific grouping of the samples based on the amount of emulsifier used. The developed PLS models had higher *R*^2^ values for oil-in-water emulsions, ranging from 0.6863 to 0.9692 for calibration, 0.5617 to 0.8740 for validation, and 0.4618 to 0.8692 for prediction, than oil-in-aqueous mint extract emulsions, with *R*^2^ values that were in range of 0.8109–0.8934 for calibration, 0.5017–0.6620, for validation and 0.5587–0.7234 for prediction. Better results were obtained for the developed nonlinear ANN models, which showed *R*^2^ values in the range of 0.9428–0.9917 for training, 0.8515–0.9294 for testing, and 0.7377–0.8533 for the validation of oil-in-water emulsions, while for oil-in-aqueous mint extract emulsions *R*^2^ values were higher, in the range of 0.9516–0.9996 for training, 0.9311–0.9994 for testing, and 0.8113–0.9995 for validation.

## 1. Introduction

The consumption of medicinal plants is inversely related to the occurrence of diseases such as several types of cancer, cardiovascular, cerebrovascular, and neurodegenerative diseases. The high presence of antioxidants in plants, in the form of bioactive compounds, represents an important basis for the health-protecting effects connected with their consumption [1]. Due to their beneficial effects, these bioactive compounds extracted from medicinal plants have been successfully incorporated into food systems [2].

In order to increase the use of extracts with compounds isolated from medicinal plants in food matrices, several technologies should be considered: nanoemulsions, nanocapsules, vapors, and edible films [2]. There is increased interest in the food industry for emulsions as delivery systems to maintain the completeness of sensitive biocompounds with the aim to improve their bioavailability, solubility, stability, and texture and the control of their release [3]. Therefore, the industrial use of emulsions has emerged in recent years with the aim to ameliorate certain product characteristics or to create completely new ones [4,5]. 

Emulsions are multiphase systems comprising two or more liquids that are insoluble in one another [6]. One phase is a continuous phase in which small droplets of another phase are dispersed [7,8]. According to the droplet size range, emulsions can be divided into macroemulsions (0.1–100 µm), microemulsions (5–100 µm), and nanoemulsions (20–200 nm) [9,10]. Emulsions typically consist of oil, water, and emulsifier [3,11]. Emulsifiers are surface-active substances (surfactants) that are used for avoiding emulsion destabilization such as flocculation, coalescence, and Ostwald ripening [12]. They are normally soluble in one phase, consisting of functional groups capable of interaction with the other phase at the interfacial surface [6]. Emulsifiers are amphiphilic molecules that are used with the aim to maintain the emulsion stability for a long period of time by preventing droplet coalescence at the liquid–liquid interface [8].

Emulsification is a process of emulsion formation; however, this process is unspontaneous and requires energy input for droplet production [13]. Conventional high-energy methods include colloid mills, high-pressure homogenizers, and sonicators [5,14,15,16]. Major obstacles of these methods are the relatively expensive equipment and the regulation of process parameters such as the temperature, applied force, and droplet size of an emulsion [5,17]. To overcome the above-mentioned limitations, the use of microfluidic technology has the potential to eliminate the existing problems. According to the literature, transferring the emulsification to a smaller scale enables the production of emulsions with controllable droplet sizes and reduces the amount of emulsifier and time consumption [18,19,20]. The microchannel geometry allows the control of shear forces; high shear forces act on a small liquid volume (several nanoliters), allowing the precise adjustment of the emulsion droplet size. The prevailing surface tension in microchannels ensures the continuous generation of droplets with the same droplet size and shape [21]. To produce monodispersed droplets using various microfluidics, different techniques have been described, including T-junctions, Y-junctions, and flow-focusing cross-junctions [22]. According to Vidovič et al. [23], the characteristics of the dispersion being dispersed as well as the working conditions and the device being utilized determine the form and distribution of the droplets. When working to generate droplets using continuously operated microfluidic devices, the size of the droplets depends on the channel geometry, channel length, shape–flow-rate ratio, and velocity. Furthermore, the droplet size also depends on the contact and injection angle as well as the surfactant addition [24]. According to Wang et al. [25], changing the contact angle between the dispersed phase and the channel wall is the most practical way to modify the droplet size. Microchannels are mostly produced form glass and poly(dimethylsiloxane), and the effect of the wettability of those materials on the contact angle has to be taken into consideration [26]. Due to the significance of the contact angle in the design of microdroplet preparation machinery as well as its role in the selection of appropriate raw materials for industrial processes, it is crucial to understand how it affects step emulsification. In experimental work, applying different surfactants frequently alters a fluid’s interfacial tension and contact angle.

Maintaining emulsion stability is one of the greatest challenges during the emulsification process. As mentioned, the mixing speed, mixing time, emulsification method, oil composition, oil-to-water ratio, emulsifier type, and concentration are parameters that influence the droplet size distribution [10,27]. Therefore, it is necessary to develop a rapid and nondestructive method for monitoring the emulsification process. According to the literature, the application of near-infrared spectroscopy (NIRs) has emerged for monitoring variables influencing the formation of emulsions [10,28,29,30]. 

Near-infrared spectroscopy (NIRs), as a nondestructive, noninvasive, fast, and efficient technique, has a long history of application in the food industry [31,32,33]. The advantages of its application include having little or no need for sample preparation; the lower cost compared to conventional analytical techniques, and the capability to analyze a wide range of products [33]. NIRs is based on the absorption of NIR radiation in the wavelength range from 780 nm to 2500 nm [34]. The absorbance of light is mainly caused by overtones and the combination vibrations of some hydrogen-based functional groups such as O–H, C–H, C–O, and N–H [24]. Due to the large number of recorded spectra, it is imperative to analyze the acquired spectral data. In order to extract important information and to identify significant patterns in NIR spectra, various mathematical and statistical methods (principal component analysis, (PCA), partial least-squares regression (PLSR), canonical correlation analysis (CCA), and principal component regression (PCR)) can be used [10,23]. The big advantage of these statistical methods includes exploring these spectral data for qualitative and quantitative applications [35]. However, due to the complex nature of food, nonlinear techniques, compared to multivariate methods, have proved to be a good solution. Artificial neural networks (ANNs) coupled with NIR spectroscopy have been identified as an excellent tool for monitoring emulsion droplet dimeter prediction [10], the prediction of the physical and chemical properties of plant extracts [36], and honey adulteration detection and quantification [37]. For all three mentioned experiments, developed ANN models described the experimental data with high accuracy (the coefficients of determination were greater than 0.8).

The aim of this work was to predict the average Feret droplet diameters of oil-in-water and oil-in-aqueous mint extract emulsions based on the combination of NIR spectra with PLS regression and ANN modeling. Emulsification was performed in a microfluidic system including a static teardrop micromixer with the addition of three emulsifiers (PEG 1500, PEG 6000, and PEG 20,000) at three different concentrations (2%, 4%, and 6%). To the best of our knowledge this is the first application of NIR spectra and PLS and ANN modeling for the analysis of oil-in-aqueous mint extract emulsions prepared using a microfluidic device, motivated by the fact that emulsion technology is generally applied for the encapsulation of bioactives in aqueous solutions, which can either be used directly in the liquid state or can be dried to form powders [38]. Different spectral preprocessing methods (first-order Savitzky–Golay derivative (SG1), standard normal variate (SNV), multiplicative scatter corrections (MSC), first-order Savitzky–Golay derivative followed by standard normal variate (SG1+SNV), and first-order Savitzky–Golay derivative followed by multiplicative scatter corrections (SG1+MSC)) were applied in order to determine the predictive ability of the models used.

## 2. Materials and Methods

### 2.1. Materials

Edible sunflower oil (Zvijezda plus d.o.o., Zagreb, Croatia) was purchased from a local supermarket. Polyethylene glycols with average molecular weights of 1500 g/mol (PEG 1500) and 6000 g/mol (PEG 6000) were purchased from Acros Organics (Geel, Belgium), while the 20,000 g/mol polyethylene glycol (PEG 20,000) was obtained from Sigma-Aldrich (Taufkirchen, Germany). Dried mint leaves (*Mentha piperita* L.) were purchased from Suban, Croatia. Plant materials were collected during the flowering season of 2019 in the north-western part of Croatia, dried naturally, and stored in ambient conditions before use.

### 2.2. Methods

#### 2.2.1. Mint Extract Preparation

First, 1 g of dry plant material was placed in a 200 mL glass with 50 mL of deionized water, and solid–liquid extraction was performed using an Ika HBR4 digital oil bath (IKA-Werk GmbH & Co., KG, Staufen, Germany) at 80 °C and 250 rpm for 30 min. After the extraction, samples were filtered through a 100% cellulose paper filter (LLG Labware, Meckenheim, Germany) with 5–13 µm pores and stored at 4 °C until analyzed. The dry matter content of the aqueous mint extract was 0.85%. 

#### 2.2.2. Emulsification in a Microfluidic System 

Glass microchips with laser-engraved microchannels were placed in stainless-steel holders, which provided leak-free connections (Micronit Microfluidics B.V., Enschede, The Netherlands). Experiments were performed in microchannels with the following dimensions: width/height/length = 250 μm: 150 μm: 55.3 mm. The microchannels were equipped with static teardrop micromixers. Emulsion droplets were generated in a borosilicate glass microfluidic device. Two syringe pumps (NE -1000 Syringe Pump, New Era Pump Systems, New York, NY, USA) with high-pressure stainless-steel syringes (8 mL, Harvard Apparatus) were used for solution delivery. Two phases, oil and aqueous mint extract, were introduced separately into microchannels through a fused silica connection (375 µm o.d., 150 µm i.d., Micronit Microfluidics B.V., Enschede, The Netherlands). The emulsification experiments were performed according to the design of experiments previously described by Grgić et al. [39] (Table 1).

#### 2.2.3. Average Feret Diameter

The prepared samples of oil-in-aqueous mint extract emulsions were photographed using a microscope equipped with a camera (BTC type LCD-35, Bresser, Germany) at 4× magnification. The average Feret diameter of the droplets was measured using the software tool ImageJ (v.1.8.0. National Institutes of Health, Bethesda, MD, USA). The Feret diameter was defined as the perpendicular distance between two tangents located on opposite sides of a particle [39]. The average Feret diameter of oil-in-water emulsions prepared in the same microfluidic system according to the same experimental design (Table 1) was previously published in an article by Grgić et al. [39]. 

#### 2.2.4. Near-Infrared Spectra of Emulsions

The near-infrared (NIR) spectra of all oil-in-water and oil-in-aqueous mint extract emulsions were recorded in the wavelength range from 904 nm to 1699 nm using an NIR spectrometer (NIR—128—1.7—USB/6.25/50 µm Control Development Inc., South Bend, IN, USA). The NIR spectra were recorded in disposable plastic cuvettes with a liquid sample measurement setup. The NIR spectra were recorded in three parallel runs.

#### 2.2.5. NIR Spectra Processing and Modeling

The effects of preprocessing methods of NIR spectra on sample grouping were analyzed using the Unscrambler X software (Version 10.1. CAMO AS, Oslo, Norway). Oil-in-water emulsions and oil-in-aqueous mint extract emulsions were analyzed separately. The performances of the following preprocessing methods were tested: (i) raw spectra, (ii) first-order Savitzky–Golay derivative (SG1), (iii) standard normal variate (SNV), (iv) multiplicative scatter corrections (MSC), (v) first-order Savitzky–Golay derivative followed by standard normal variate (SG1+SNV), and (vi) first-order Savitzky–Golay derivative followed by multiplicative scatter corrections (SG1+MSC). The same software was also used for a principal component analysis (PCA) and a partial least-squares (PLS) regression. 

For the prediction of the average droplet sizes of the oil-in-water emulsions and oil-in-aqueous mint extract emulsions with PEG 1500, PEG 6000, or PEG 20,000 as emulsifiers, PLS regression models were developed. The PLS model input data were the triplicates of the spectra of each sample. Each PLS model used seven latent variables (factors) and a random cross-validation method based on splitting the input data into 20 segments. No normalization method was used before the data transformation. The applicability of the PLS models developed using raw and preprocessed NIR spectra was estimated based on: (i) the coefficients of determination for calibration (*R*^2^_cal_) and cross-validation (*R*^2^_cval_), (ii) the root-mean-square error for calibration (RMSEC) and cross-validation (RMSECV), (iii) the average value of the difference between the predicted and observed values (bias), and (iv) the ratio of the predicted deviation (RPD) and the range error ratio (RER) [10,37].

**Figure 1:**
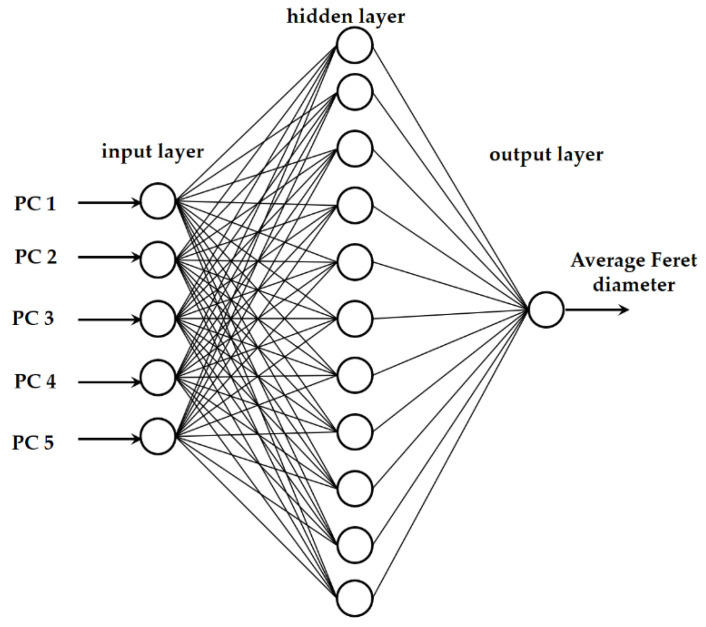
Graphical representation of the neural network model.

The average droplet sizes of the oil-in-water emulsions and oil-in-aqueous mint extract emulsions with PEG 1500, PEG 6000, or PEG 20,000 as an emulsifier based on NIR spectra were also predicted using artificial neural network (ANN) modeling in Statistica v.13.0 software (Tibco Software Inc., Tulsa, OT, USA). Multiple layer perceptron network (MLP network) models consisted of an input layer, a hidden layer, and an output layer (Figure 1). The first five factors from the PCA analysis were represented by the five neurons in the input layer. ANN inputs were chosen from the first five principal components, which accounted for more than 99.99% of the data variability. A PCA based on the raw spectra and a PCA based on the selected preprocessing method were used separately. The following set of alternatives was randomly chosen as the hidden activation function and the output activation function: identity, logistic, hyperbolic tangent, and exponential. The MLP chose a random number between 3 and 11 neurons for the hidden layer. 

The data matrix for ANN modeling comprised 51 rows representing emulsion samples prepared using an individual emulsifier and 6 columns referring to five PCA coordinates (factors) and the measured average droplet sizes. During the construction, data were randomly divided into 70% for network training, 15% for network testing, and 15% for model validation. For each emulsifier, 1000 networks were generated. Model training was carried out using a back error propagation algorithm and the sum-of-squares error function implemented in Statistica v.13.0 (Tibco Software Inc., Tulsa, OT, USA) automated neural networks. The proposed ANN model performance was estimated based on the *R*^2^ and root-mean-square error (RMSE) values for the training, testing, and validation.

## 3. Results and Discussion 

### 3.1. The Average Feret Diameters of Oil-in-Aqueous Mint Emulsions: Comparison with the Average Feret Diameters of Oil-in-Water Emulsions

**Figure 2:**
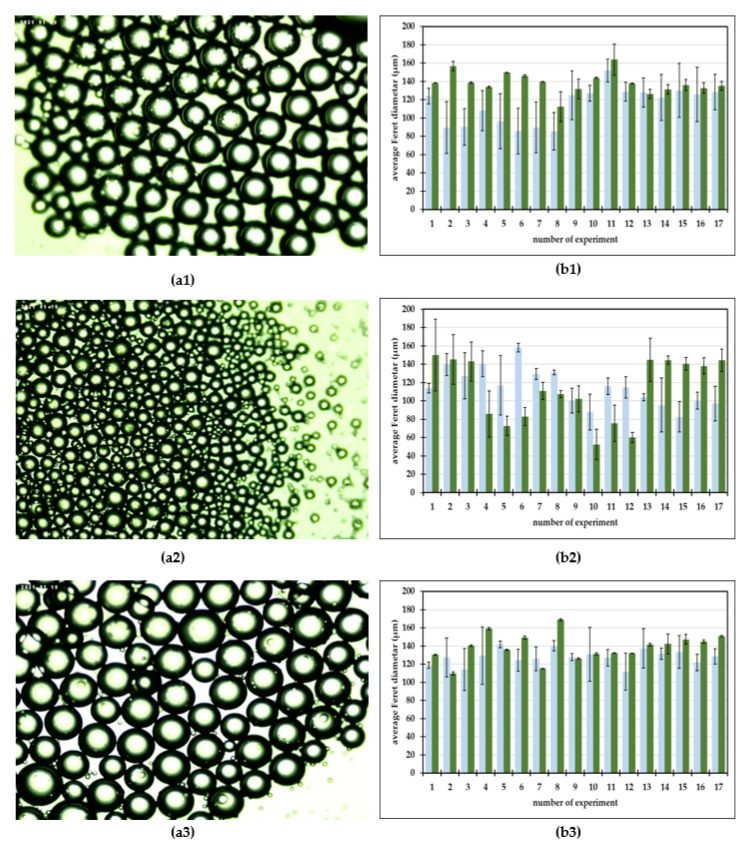
(**a**) Photos of oil-in-aqueous mint extract emulsions. (**b**) Average Feret diameters of oil-in-water emulsions (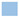) and oil-in-aqueous mint extract emulsions (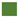). (**a1**,**b1**) PEG 1500, (**a2**,**b2**) PEG 6000, (**a3**,**b3**) PEG 20,000.

This research examined the applicability of a microfluidic device for generating oil–aqueous mint extract emulsions utilizing emulsifiers such as PEG 1500, PEG 6000, and PEG 20,000. The prepared emulsions were observed under a microscope at the microfluidic device outlet. Photos were taken and used for the average Feret diameter measurements. The gathered photos of the prepared emulsions and measured average Feret diameters are presented in Figure 2.

It can be observed that spherical oil droplets of a dispersed phase were generated in the continuous aqueous mint extract (Figure 2(a1–a3)). As previously described by Shah et al. [40], in contrast to bulk emulsification techniques, an emulsion is carefully manufactured one drop at a time in a microfluidic device, and therefore a monodisperse emulsion is the end product of this procedure. A microfluidic device’s ability to create controlled-sized emulsion [41,42] droplets depends on a number of factors, including the flow rates, the fluid viscosity, the emulsifier, and the geometry of the microfluidic channels [10,34]. All of the mentioned factors have to be taken into consideration when optimizing the emulsification process [39]. 

For oil-in-aqueous mint extract emulsions, the smallest droplets were generated with the emulsifier PEG 6000 (Figure 2(a2,b2)), with an average Feret dimeter in the range from 52.36 ± 16.50 µm to 149.99 ± 39.15 µm, followed by the emulsions with PEG 1500 (Figure 2(a1,b1)) and with PEG 20,000 (Figure 2(a3,b3)). For PEG 1500 emulsions, the average droplet Feret dimeter was in the range from 112.26 ± 16.24 µm to 163.77 ± 16.93 µm, while for the PEG 20,000 emulsions the average droplet Feret dimeter was in the range from 109.81 ± 1.74 µm to 169.99 ± 1.06 µm. When comparing the measured data with the results presented by Grgić et al. [39] for oil-in-water emulsions produced with the same emulsifiers and using the same process conditions, the droplet size followed approximately the same trend. For PEG 1500 and PEG 20,000 oil-in-mint extracts, the emulsion droplets were larger than the oil-in-water emulsion droplets for all experiments. For PEG 6000 oil-in-mint extracts, the emulsion droplets were larger than the oil-in-water emulsion droplets for experiments with 30% oil phase.

### 3.2. NIR Spectra of Oil-in-Water and Oil-in-Aqueous Mint Extract Emulsions: Preprocessing and PCA Analysis

**Figure 3:**
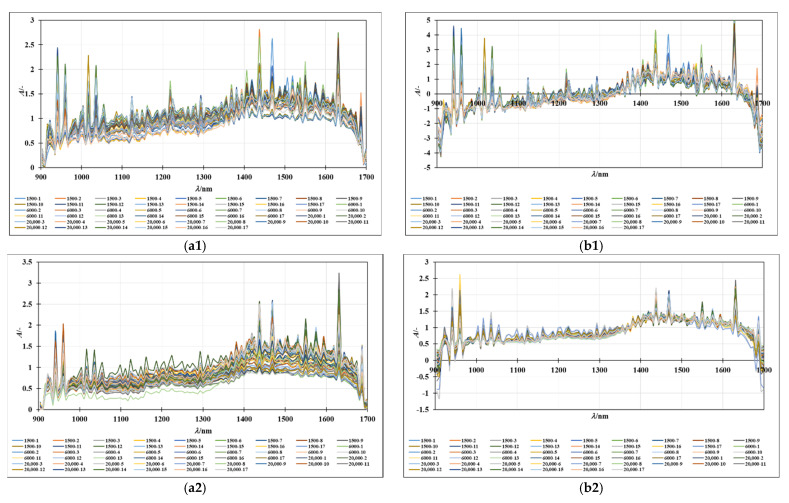
(**a**) Raw spectra of (**a1**) oil-in-water and (**a2**) oil-in-mint aqueous extracts. (**b**) (**b1**) SNV preprocessed spectra of oil-in-water emulsions and (**b2**) MSC preprocessed spectra of oil-in-mint aqueous extract emulsions.

The NIR spectra of the oil-in-water and oil-in-aqueous mint extract emulsions prepared with different emulsifiers (PEG 1500, PEG 6000, and PEG 20,000) were recorded continuously in the wavelength range from 904 to 1699 nm. The average spectra of individual samples, grouped according to the continuous phase, are given in Figure 3(a1,a2), while the SNV preprocessed spectra of the oil-in-water emulsions and the MSC preprocessed spectra of oil-in-mint aqueous extract emulsions are given in Figure 3(b1,b2). Despite variations in spectral absorbance, the majority of the spectra obtained from the emulsion samples followed a similar pattern. Additive effects (spectral shifts) characteristic of the different droplet sizes present in the samples can be seen. A similar observation was made in a study by Bampi et al. [28], where a discrepancy in the spectral baseline was observed that could be attributed to different light scattering due to the different water droplet sizes in the emulsions. The largest differences in the spectral peaks of both sample types are seen for the wavelength range from 1300 to 1699 nm, which is specific for the superposition of the O-H bonds. Moreover, the differences in this part of the spectrum can be easily correlated with the water present in the samples [36]. 

**Figure 4:**
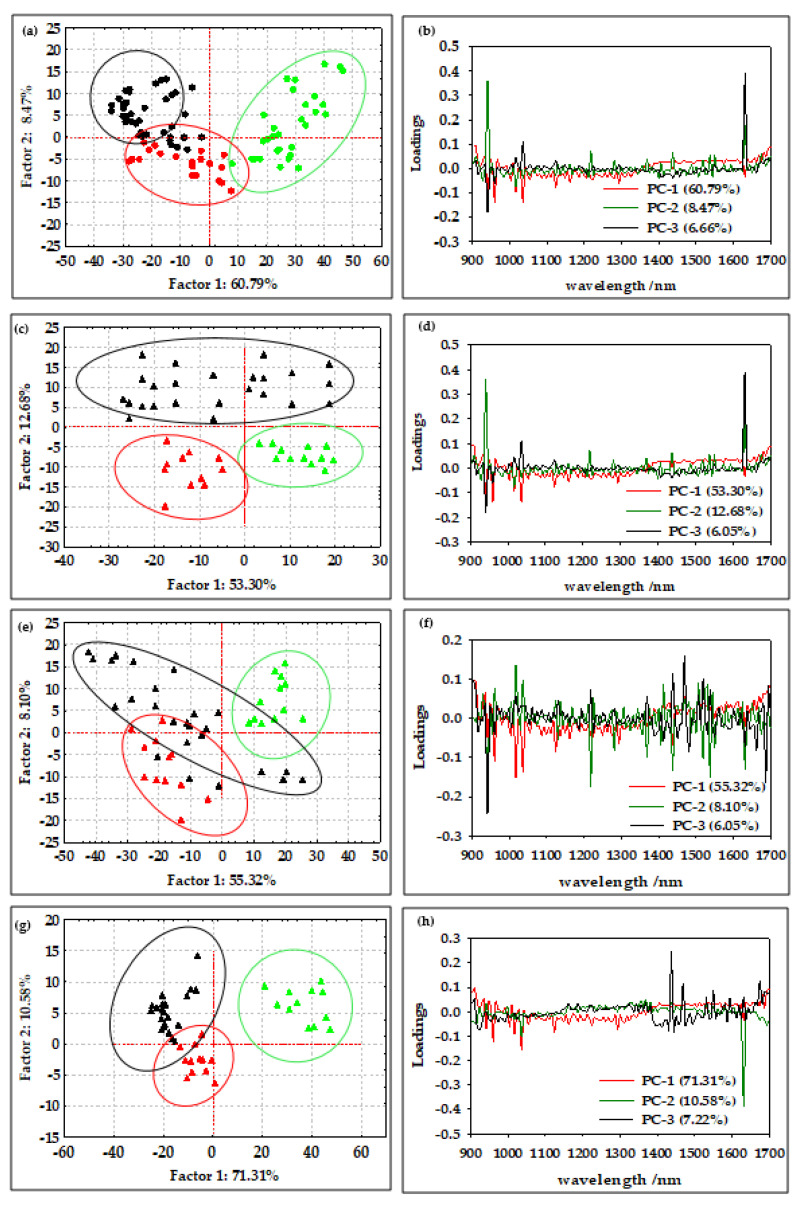
(**a**) PCA score plot of all average oil-in-water emulsion NIR spectra after SNV preprocessing (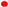 PEG 1500, 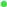 PEG 6000, 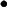 PEG 20,000). (**b**) PCA loading of all average oil-in-water emulsion NIR spectra after SNV preprocessing. PCA score plot of NIR spectra of oil-in-water emulsion NIR spectra after SNV preprocessing with (**c**) PEG 1500, (**e**) PEG 6000, and (**g**) PEG 20,000 (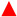 2% emulsifier, 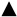 4% emulsifier, 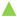 6% emulsifier). PCA loading of NIR spectra of oil-in water emulsion NIR spectra after SNV preprocessing with (**d**) PEG 1500, (**f**) PEG 6000, and (**h**) PEG 20,000.

**Figure 5:**
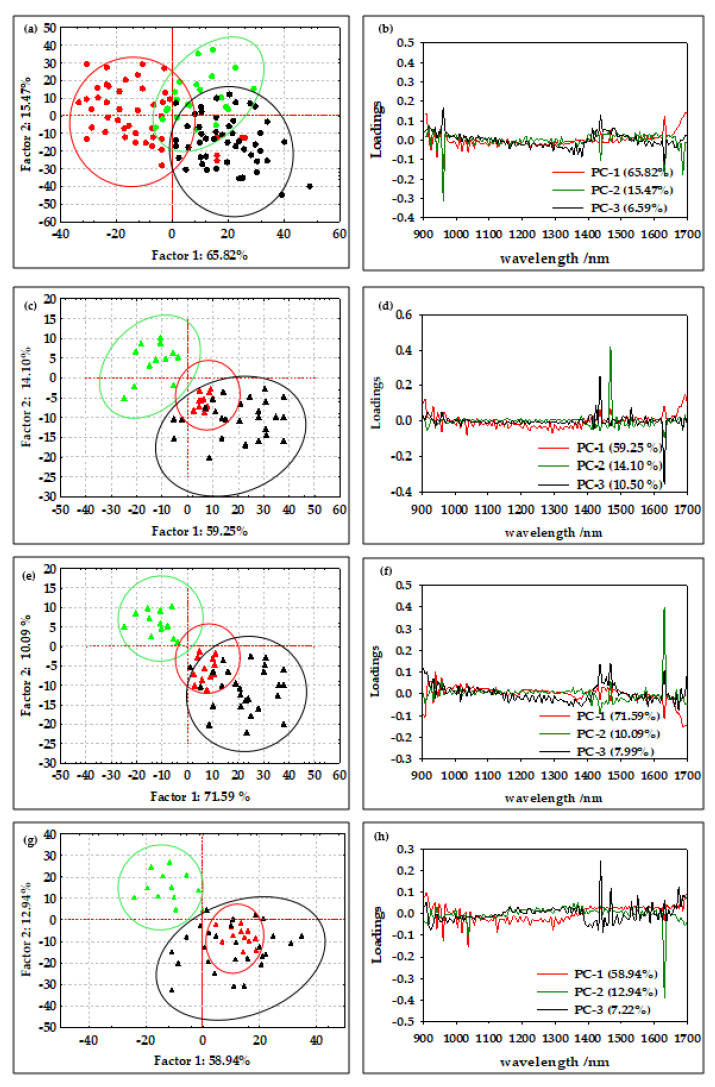
(**a**) PCA score plot of all average oil-in-aqueous mint extract emulsion NIR spectra after MSC preprocessing (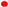 PEG 1500, 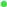 PEG 6000, 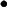 PEG 20,000). (**b**) PCA loading of all average oil-in-aqueous mint extract emulsion NIR spectra after MSC preprocessing. PCA score plot of NIR spectra of oil-in-aqueous mint extract emulsion NIR spectra after SNV preprocessing with (**c**) PEG 1500, (**e**) PEG 6000, and (**g**) PEG 20,000 (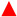 2% emulsifier, 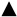 4% emulsifier, 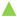 6% emulsifier). PCA loading of NIR spectra of oil-in-aqueous mint extract emulsion NIR spectra after SNV preprocessing with (**d**) PEG 1500, (**f**) PEG 6000, and (**h**) PEG 20,000.

According to Borges et al. [29], NIR spectroscopy is a method that allows the efficient determination of the average diameter and water content of oil-in-water emulsions and offers great potential for the online qualitative analysis of biodiesel during storage. Furthermore, according to Bi et al. [43] and Grisanti et al. [44], the implication that numerous interferences frequently cause spectra to be altered during the signal acquisition process is a practical issue for the implementation of NIR technology. The sample thickness, measurement geometry, or physical characteristics of the samples can influence the light path length, and therefore preprocessing is an essential step in NIR spectral modeling [45]. As described by Feng et al. [46], spectral preprocessing is used to eliminate systemic noise and highlight the changes between the samples. In this work, the efficiency of the first-order Savitzky–Golay derivative (SG1), standard normal variate (SNV), multiplicative scatter corrections (MSC), first-order Savitzky–Golay derivative followed by standard normal variate (SG1+SNV), and first-order Savitzky–Golay derivative followed by multiplicative scatter corrections (SG1+MSC) was analyzed. The results showed that SNV and MSC were the most efficient methods for the analysis of the NIR spectra of oil-in-water and oil-in-aqueous mint extract emulsions prepared in a continuously operated microfluidic device. The standard normal variate (SNV) and multiplicative signal correction (MSC) methods was analyzed (Figure 3(b1,b2)). By deducting the complete spectrum’s mean value, the standard normal variate (SNV) eliminates a constant offset component and scales down all spectra by dividing the result by the full spectrum’s standard deviation [47]. On the other hand, by employing the linear least-squares approach to construct a linear model between a reference spectrum and other spectra in the dataset, the multiplicative scatter correction reduces spectral deviations. The dataset’s average spectrum, known as the reference spectrum, is frequently selected [48]. However, without the application of chemometric methods that can extract significant data from the spectra, few conclusions can be drawn from either the raw or preprocessed spectral data. For this reason, a PCA analysis was applied to the raw spectral data and preprocessed data as one of the most widely used chemometric methods to detect the differences between samples [10]. The results for the oil-in-water emulsions are shown in Figure 4, and the results for the oil-in-aqueous mint extract emulsions are shown in Figure 5. Based on the PCA results, SNV was selected as the optimal preprocessing method for individual oil-in-water emulsion NIR spectra and MSC was selected as the optimal preprocessing method for individual oil-in-aqueous mint extract emulsion NIR spectra. The selected preprocessing methods resulted in the best discrimination of the sample and the highest explanation of the data in the first two principal components [49]. PCA score plots for average NIR preprocessed spectra (Figure 4a and Figure 5a) show the specific grouping of the samples in three groups according to the emulsifier used (PEG 1500, PEG 6000, and PEG 20,000). As expected, the grouping was more evident for the oil-in-water emulsions. Mint extracts, as the continuous phase for the second type of emulsion, scarcely influenced the sample grouping. The chart of the principal components (*x*-axis) and the percentage of explained variance (*y*-axis) shows its inflection point at the third PC, which is an indication of the most important PCs to investigate the observed system. As presented for both types of emulsions, the first three principal components (PCs) explained most of the sample variability. For the oil-in-water emulsions, the first three PCs explained 75.92% of the data variability, while for the oil-in-aqueous mint extract emulsions the first three PCs explained 87.06% of the data variability. The compressed variance difference in PC1–PC3 for the oil-in-water emulsions and oil-in-aqueous mint extract emulsions could be explained by the color difference of the continuous phases used and the larger effect of color in PC1–PC3 of the oil-in-aqueous mint extract emulsions. The detection of significant variables (variables with large variances) and correlations between variables [50] was made by the analysis of the loading spectra (Figure 4b and Figure 5b). Even though both positive and negative contributions are displayed in the loading plots (Figure 4b and Figure 5b), the spectral shape of the pure PC1 loading vector displays the majority of the distinctive absorption peaks observed in Figure 2a and Figure 3a. Moreover, the intensity maximum of the pure PC1 spectrum is shifted toward the actual spectrum by the positive and negative contributions from PC2 and PC3, as previously presented by Zhang et al. [51]. For the oil-in-water emulsions, the maximum loading peaks were noticed at 941 nm (C−H bond, 3rd overtone) and 1631 nm (C−H, 1st overtone), while for the oil-in-aqueous mint extract emulsions the maximum loading peaks were noticed at 960 nm (C−H bond, 3rd overtone), 1437 nm (O−H, 1st overtone), and 1631 nm (C−H, 1st overtone). Furthermore, a PCA analysis was applied for the analysis of the emulsion samples according to the emulsifier used. As presented in Figure 4c,e,g and Figure 5c,e,g, the specific grouping of the samples based on the amount of the emulsifier used can be noticed. It can be seen that sample grouping was more pronounced for the oil-in-water emulsions. Furthermore, the emulsions with the highest amount of emulsifier (6%) were specifically grouped, while there was some overlap of the samples with 2% and 4% emulsifier. That was especially evident for the oil-in-aqueous mint extract emulsions (Figure 5c,e,g). Moreover, the first three factors explained 75.31% (PEG 1500), 89.11% (PEG 6000), and 89.11% (PEG 20,000) of the variability between the oil-in-water emulsion samples and 83.85% (PEG 1500), 89.24% (PEG 6000), and 96.23% (PEG 20,000) of the variability between the oil-in-aqueous mint extract emulsion samples. The obtained results indicate that those samples were also separated based on the mixing rate, oil phase, and particle size. The loading plot shows that for oil-in-water emulsions the maximum loading peaks were noticed as follows: (i) for PEG1500 at 941 nm (C−H bond, 3rd overtone) and 1631 nm (C−H, 1st overtone) (Figure 4d); (ii) for PEG 6000 at 941 nm (C-H bond, 3rd overtone), 1218 nm (C−H, 2nd overtone), and 1687 nm (C−H, 1st overtone) (Figure 4f); and (iii) for PEG 20,000 at 1631 nm (C−H, 1st overtone) (Figure 4h). Moreover, for the oil-in-aqueous mint extract emulsions, maximum loading peaks were noticed as follows: (i) for PEG1500 at 1469 nm (O−H, 1st overtone) and 1631 nm(C−H, 1st overtone) (Figure 5d); (ii) for PEG 6000 at 1438 nm (O−H, 1st overtone), 1469 nm (O−H, 1st overtone), and 1631 nm (C−H, 1st overtone) (Figure 5f); and (iii) for PEG 20000 at 960 nm (C−H bond, 3rd overtone), 1437 nm (O−H, 1st overtone), 1631 nm (C−H, 1st overtone), and 1687 nm (C−H, 1st overtone) (Figure 5h). The high percentages of explained variance in both cases are indicative that NIR coupled with chemometrics can be successfully used for the rapid and nondestructive discrimination of emulsion samples. These results are consistent with studies by Borges et al. [29], Bampi et al. [28], and Dinache et al. [52], who also successfully used NIR spectroscopy coupled with chemometrics to analyze emulsions.

### 3.3. PLS Modeling of the Average Feret Diameters of Emulsions

The aim of this work was to analyze the applicability of NIR spectroscopy to distinguishing the emulsion droplet sizes, expressed as the average Feret diameter at the microfluidics device outflow. For that purpose, partial least-squares (PLS) modeling was applied (Table 2).

The performances of the different preprocessing methods were tested, including the first-order Savitzky–Golay derivative, standard normal variate, multiplicative scatter corrections, first-order Savitzky–Golay derivative in combination with standard normal variate, and first-order Savitzky–Golay derivative in combination with multiplicative scatter corrections. The applicability of the developed PLS models was evaluated using *R*^2^_cal_, *R*^2^_cval_, RMSEC, RMSECV, bias, RPD, and RER. As for the PCA analysis, the SNV preprocessing method was shown to be the most efficient for the PLS modeling using the NIR spectra of the oil-in-water emulsions, whereas the MSC was the most efficient for the PLS modeling using the NIR spectra of the oil-in-aqueous mint extract emulsions.

**Figure 6:**
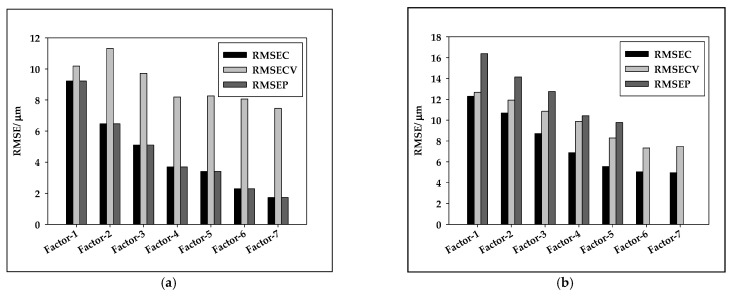
Variation in RMSEC, RMSECV, and RMSEP as a function of the latent variable number for the best PLS models for (**a**) oil-in-water emulsions and (**b**) oil-in-mint extract emulsions.

From the results summarized in Table 2 and Figure 6, it can be seen that the PLS model for oil-in-water emulsions using SNV preprocessed NIR spectra showed strong correlations (*R*^2^ > 0.7) [53] for the coefficient of calibration (*R*^2^_cal_), cross-validation (*R*^2^_cval_), and prediction (*R*^2^_pred_) with the addition of PEG 20000 (*R*^2^_cal_ of 0.9692, *R*^2^_cval_ of 0.8740, and *R*^2^_pred_ of 0.8692). For PEG 15000, the PLS model correlation was moderate (*R*^2^_cal_ of 0.6863, *R*^2^_cval_ of 0.5617, and *R*^2^_pred_ of 0.4618). For PEG 6000, a strong correlation was achieved for the coefficient of calibration (*R*^2^_cal_ of 0.9601), while for cross-validation and prediction the correlations were moderate (*R*^2^_cval_ of 0.6204 and *R*^2^_pred_ of 0.6254). It can also be noticed that there were variations in RMSEC, RMSECV, and RMSEP for a number of PLS model factors. The results indicate that PLS models for oil-in-aqueous mint extract emulsions showed lower accuracy in comparison to those for oil-in-water emulsions. The highest *R*^2^_pred_ of 0.7234 was obtained for emulsions prepared with PEG 1500, followed by PEG 20000, with an *R*^2^_pred_ of 0.7062, and PEG 6000, with an *R*^2^_pred_ of 0.5587. These poor values can be attributed to the indirect measurement of the average Feret diameter [54]. The quality of the developed PLS models was also evaluated based on residual predictive deviation (RPD) index and the range-to-error ratio (RER). Ideal and robust models need to possess higher *R*^2^_pred_ coefficients and RPD indexes [55]. Based on the obtained RPD values, the only model for oil-in-water emulsion with PEG 20000 with RPD = 7.4581 was the one that can be used for process control (RPD > 6.5) [56]. The other developed PLS models, except the model for oil-in-aqueous mint extract emulsions with PEG 6000, can be used for screening (1.5 < RPD < 2.5) [57]. The bias, which is the discrepancy between the means of the true values and the estimated values, additionally known as the error of means, was strongly affected by the measurement error and the number of predictor variables [58]. Comparing the developed PLS models, it could be noticed that lower bias values were obtained for the oil-in-mint aqueous extract emulsions. 

The efficient use of NIR spectroscopy coupled with PLS modeling was presented by Mishra et al. [59] for the at-line and in-line monitoring of droplet size in mayonnaise. The authors developed PLS models that achieved prediction errors for droplets in the range of 0.38 to 0.68 µm. Moreover, Amsaraj et al. [60] combined Fourier-transform infrared spectroscopy with a partial least-squares discriminant analysis of milk adulteration and achieved a 100% accurate classification. Bampi et al. [28] proposed PLS models with 9.53% mean error for the external validation of the average droplet size of water–biodiesel emulsions, while Jurinjak Tušek et al. [10] applied PLS modeling to predict the average Feret diameter of oil-in-water emulsions with two different emulsifiers (Tween 20 and PEG 2000). 

### 3.4. ANN Modeling of the Average Feret Diameters of Emulsions

A multilayer feed-forward neural network or multilayer perceptron (MLP) was fitted using the training dataset of the average droplet Feret diameter of each individual produced emulsion. The model inputs were the first five PCs, which contributed 99% of the data variability selected after the preprocessing that ensured efficient sample grouping. For the oil-in-water emulsions, five PCs after SNV preprocessing were used, and for the oil-in-aqueous mint extract emulsions, five PCs after MSC preprocessing were used, as in the case of PLS modeling. The performances of selected ANNs are given in Table 3 and in Figure 6. Based on the obtained *R*^2^ and RMSE values for training, testing, and validation, it can be noticed that ANN modeling ensured better agreement between the experimental data and the model-predicted data than PLS modeling. This can be simply explained by the nature of the model; PLS models include linear regressions, while ANN models include highly nonlinear expressions. 

**Figure 7:**
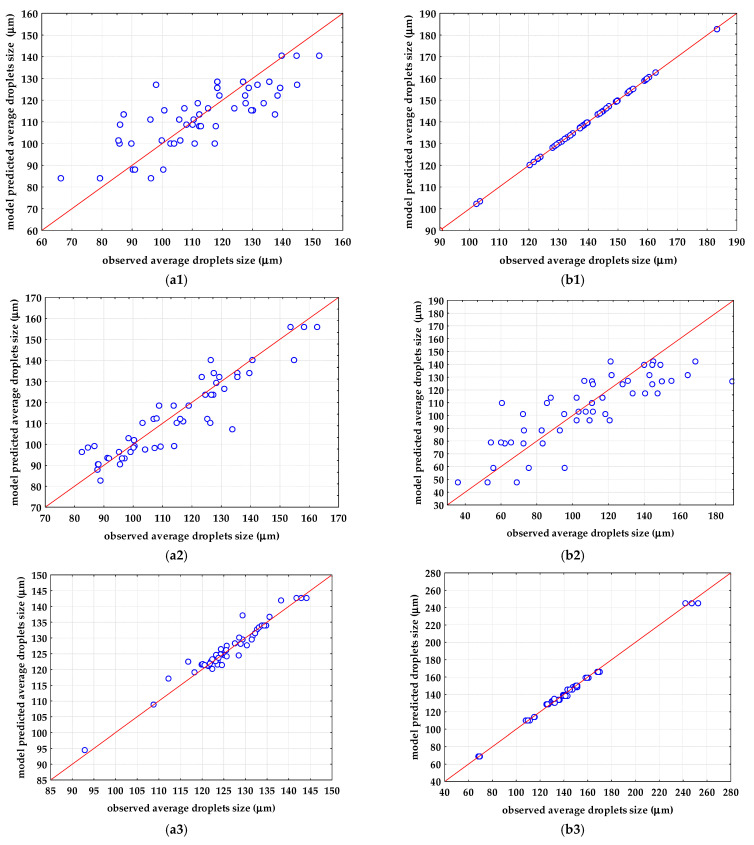
Comparisons between observations and ANN model predictions for the average droplets sizes of (**a**) oil-in-water emulsions and (**b**) oil-in-aqueous mint extract emulsions: (**a1**,**b1**) PEG 1500, (**a2**,**b2**) PEG 6000, (**a3**,**b3**) PEG 20,000.

The optimal ANN architecture was selected based on the number of neurons in the hidden layer (less neurons in the hidden layer means a simpler and more stable network). For the oil-in-water emulsions, the highest *R*^2^_validation_ was obtained for the emulsion with PEG 20000 (Figure 7(a3)). For the prediction of the average droplet Feret diameters in the oil-in-water emulsion with PEG 20000, MLP 5-5-1 was selected. The selected ANN was characterized by five neurons in the input layer, five neurons in the hidden layer, and one neuron in the output layer. A hidden activation function and output activation function was the exponential function. MLP 5-5-1 achieved an *R*^2^_training_ of 0.9917, an RMSE_training_ of 0.0002, an *R*^2^_test_ of 0.9294, an RMSE_training_ of 0.00184, an *R*^2^_validation_ of 0.8533, and an RMSE_validation_ of 0.0027. The ANN models used for the description of the average droplet Feret diameter of oil-in-aqueous mint extract emulsions showed significant improvement regarding *R*^2^_validation_ in comparison to the developed PLS models. For oil-in-aqueous mint extract emulsions, the highest *R*^2^_validation_ was obtained for emulsions with PEG 1500 (Figure 7(b1)) using MLP 5-8-1 with five neurons in the input layer, eight neurons in the hidden layer, and one neuron in the output layer. The selected ANN included a logistic function as a hidden activation function and an identity function as an output activation function. This ANN achieved an *R*^2^_training_ of 0.9996, an RMSE_training_ of 0.0004, an *R*^2^_test_ of 0.9994, an RMSE_training_ of 0.0006, an *R*^2^_validation_ of 0.9995, and an RMSE_validation_ of 0.0005. 

The effectiveness of ANN modeling vs PLS modeling was estimated using R^2^ and RMSE, and based on those criteria, the developed ANN models showed higher effectiveness for the prediction of the average Feret diameter of both oil-in-water and oil in mint aqueous emulsions. However, the proposed models were limited in their applicability to the range of the trained variables and therefore do not have a wider application. As previously described, the superior performance of the ANN model is attributed to its nonlinear mapping capability, which is a feature lacking in the PLS models [61,62], and it is in agreement with previously presented results where ANN modeling was also applied for the prediction of emulsion droplets using NIR spectra [10,28]. Furthermore, ANN modeling was shown to be more efficient than PLS modeling for the rapid detection of the microbial spoilage of beef fillets based on Fourier-transform infrared spectra [63], for the prediction of soil organic carbon using UV-VIS-NIR spectra [64], for the quantitative analysis of quartz in the presence of mineral interferences using FTIR spectra [62], for nondestructive grape texture prediction using NIR spectra [49], and for the prediction of bioactive component contents in olive leaf extracts using NIR spectra [36].

## 4. Conclusions

This is the first application of NIR spectra and PLS and ANN modeling for the analysis of oil-in-aqueous mint extract emulsions prepared using a microfluidic device. Based on the presented results, it can be concluded that, for all emulsifiers used, oil-in-aqueous mint extract emulsion droplets were larger than those generated in oil-in-water emulsions. The results also showed that NIR spectroscopy coupled with chemometrics can be used for the distinctive qualitative and quantitative grouping of the samples according to the emulsifier used for its production. The obtained results for the ANN models a showed higher ability to predict the average droplet sizes than the PLS models, especially in case of PEG 1500 oil-in-aqueous mint extract emulsions, where *R*^2^ values of 0.9996, 0.9994, and 0.9995 were obtained for training, testing, and validation, respectively. This was attributed to the ANN’s nonlinear mapping capability, which is a feature lacking in the examined PLS models. 

## Figures and Tables

**Table 1:** Design of emulsification experiments. Levels of each input variable are given in brackets (−1, 1, and 0: low, medium, and high levels).

Exp.	Emulsifier Concentration (%)	Oil Concentration (%)	Total Flow Rate (µL/min)
1.	2 (−1)	30 (0)	200 (−1)
2.	6 (1)	30 (0)	200 (−1)
3.	2 (−1)	30 (0)	400 (1)
4.	6 (1)	30 (0)	400 (1)
5.	2 (−1)	25 (−1)	300 (0)
6.	6 (1)	25 (−1)	300 (0)
7.	2 (−1)	35 (1)	300 (0)
8.	6 (1)	35 (1)	300 (0)
9.	4 (0)	25 (−1)	200 (−1)
10.	4 (0)	25 (−1)	400 (1)
11.	4 (0)	35 (1)	200 (−1)
12.	4 (0)	35 (1)	400 (1)
13.	4 (0)	30 (0)	300 (0)
14.	4 (0)	30 (0)	300 (0)
15.	4 (0)	30 (0)	300 (0)
16.	4 (0)	30 (0)	300 (0)
17.	4 (0)	30 (0)	300 (0)

**Table 2:** Parameters of partial least-squares (PLS) models for the prediction of the average droplet size, expressed as the average Feret diameter of oil-in-water emulsions and oil-in-aqueous mint extract (green shading) emulsions prepared in a microfluidic device at different flow rates using different emulsifiers based on different NIR spectra pretreatments. Preprocessing methods: raw spectra (No), first-order Savitzky–Golay derivative (SG1), standard normal variate (SNV), multiplicative scatter corrections (MSC), first-order Savitzky–Golay derivative followed by standard normal variate (SG1+SNV), and first-order Savitzky–Golay derivative followed by multiplicative scatter corrections (SG1+MSC). Model applicability: the coefficients of determination for calibration (*R*^2^_cal_) and cross-validation (*R*^2^_cval_), the root-mean-square error for calibration (RMSEC) and cross-validation (RMSECV), the average value of the difference between the predicted and observed values (bias), the ratio of predicted deviation (RPD), and the range error ratio (RER). Seven factors were included in each model.

Emulsifier	Pretreatment	*R* ^2^_cal_	RSEC	*R* ^2^_val_	RMSECV	*R* ^2^_pred_	RMSEP	Bias	RPD	RER
PEG 1500	No	0.6900	11.3085	0.5496	14.5378	0.5357	13.5556	6.2689	1.6265	9.2493
	SG1	0.5803	13.1587	0.4589	15.6916	0.3509	14.5654	5.2033	1.5138	8.6081
	**SNV**	**0.6863**	**11.3765**	**0.5617**	**13.4810**	**0.4618**	**13.4267**	**6.8044**	**1.6421**	**9.3381**
	MSC	0.6891	11.4375	0.5154	14.2484	0.4582	14.8875	6.8439	1.4810	8.4218
	SG1-SNV	0.6105	12.6752	0.4871	15.2877	0.3640	16.9920	1.1515	1.2976	7.3788
	SG1-MSC	0.6127	12.6396	0.4479	15.2402	0.2065	18.5027	1.6547	1.1916	6.7763
PEG 6000	No	0.9467	4.6815	0.5865	13.2891	0.5874	13.0513	2.2884	1.6805	7.4023
	SG	0.9575	4.1833	0.7304	11.9565	0.4831	14.8774	1.5755	1.4742	6.4937
	**SNV**	**0.9601**	**4.0536**	**0.6204**	**13.0093**	**0.6254**	**12.4360**	**2.5424**	**1.7636**	**7.7686**
	MSC	0.9587	4.1230	0.6239	12.6438	0.5862	13.0704	2.2782	1.6780	7.3915
	SG1-SNV	0.9567	1.0402	0.8733	2.7853	0.6105	13.2121	2.4787	1.6600	7.3122
	SG1-MSC	0.9459	4.7151	0.6572	12.0521	0.6915	14.8931	2.1356	1.4727	6.4869
PEG 20,000	No	0.9766	1.5039	0.5695	7.1978	0.5534	5.2628	4.8615	2.4455	13.2401
	SG1	0.7898	4.5089	0.3751	8.3623	0.3963	11.5998	1.8105	1.1095	6.0070
	**SNV**	**0.9692**	**1.757**	**0.8740**	**7.4639**	**0.8692**	**1.7257**	**1.7257**	**7.4581**	**9.3778**
	MSC	0.9682	1.7532	0.4821	7.2293	0.5117	6.1176	2.7696	2.1038	11.3901
	SG1-SNV	0.9991	0.3024	0.9973	0.5394	0.9165	1.8562	2.8638	6.9337	37.5391
	SG1-MSC	0.7853	4.5565	0.3304	8.4233	0.3808	11.7133	1.1713	1.0988	5.9488
PEG 1500	No	0.8412	4.8959	0.6454	7.5579	0.6901	10.2962	0.4921	1.4379	7.8740
	SG1	0.8296	5.0706	0.6613	8.0253	0.4400	17.1542	4.1051	0.8631	4.7261
	SNV	0.8385	4.9369	0.6262	7.2036	0.7261	9.7119	0.4814	1.5244	8.2477
	**MSC**	**0.8319**	**5.0366**	**0.6620**	**7.3342**	**0.7234**	**9.7707**	**0.4076**	**1.5153**	**8.2975**
	SG1-SNV	0.8385	4.9369	0.6221	7.2793	0.7261	9.7119	0.4814	1.5244	8.1477
	SG1-MSC	0.8317	5.0404	0.5182	8.6245	0.4607	19.9587	7.4658	0.7418	4.0620
PEG 6000	No	0.8931	9.3504	0.4184	22.1524	0.5524	27.0695	1.9972	1.3227	5.6627
	SG1	0.8661	10.4682	0.1864	25.5765	0.1678	41.4123	6.4073	0.8646	3.7015
	SNV	0.8932	9.3476	0.5232	21.0092	0.5576	26.8832	2.1530	1.3319	5.7019
	**MSC**	**0.8934**	**9.3411**	**0.5017**	**20.5099**	**0.5587**	**26.8457**	**2.1110**	**1.3337**	**5.7099**
	SG1-SNV	0.8802	9.9017	0.2595	23.7959	0.1396	48.6993	6.5399	0.7352	3.1476
	SG1-MSC	0.9034	8.8882	0.2949	24.0148	0.2087	48.2772	5.8181	0.7417	3.1751
PEG 20,000	**No**	0.5648	23.0535	0.3161	31.0397	0.2218	29.6369	5.7822	1.1706	6.2226
	SG	0.6155	21.6689	0.2418	31.2997	0.2004	33.8383	3.9401	1.0253	5.4500
	SNV	0.5891	22.4006	0.3106	29.2811	0.2393	29.3017	6.0420	1.1840	6.2938
	**MSC**	**0.8109**	**15.1972**	**0.5123**	**24.1765**	**0.7062**	**18.6126**	**3.5765**	**1.8640**	**9.9083**
	SG-SNV	0.7916	15.9512	0.4772	30.3806	0.1364	41.0687	8.9847	0.8448	4.4905
	SG-MSC	0.9894	3.5941	0.4334	31.4240	0.1064	44.1265	9.0842	0.7862	4.1793

**Table 3:** Characteristics of ANN networks selected for the prediction of the average droplet sizes of oil-in-water and oil-in-aqueous mint extract (green shading) emulsions prepared in a microfluidic device at different flow rates using different emulsifiers based on different NIR spectra pretreatments.

Emulsifier/ Pretreatment	MLP	Training Perf./ Training Error	Test Perf./ Test Error	Validation Perf./ Validation Error	Hidden Activation	Output Activation
PEG 1500/ SNV	MLP 5-10-1	0.9843 0.0073	0.8085 0.0101	0.7443 0.0128	Identity	Exponential
	MLP 5-9-1	0.9844 0.0041	0.8496 0.0100	0.7364 0.0161	Identity	Identity
	MLP 5-10-1	0.9828 0.0058	0.8508 0.0125	0.7348 0.0172	Exponential	Exponential
	**MLP 5-8-1**	**0.9836** **0.0072**	**0.8515** **0.0079**	**0.7615** **0.0121**	**Logistic**	**Identity**
	MLP 5-5-1	0.9835 0.0032	0.8115 0.0120	0.7358 0.0166	Exponential	Exponential
PEG 6000/ SNV	MLP 5-4-1	0.9374 0.0048	0.9083 0.0064	0.7160 0.0074	Tanh	Exponential
	**MLP 5-8-1**	**0.9428** **0.0044**	**0.8675** **0.0044**	**0.7377** **0.0119**	**Exponential**	**Identity**
	MLP 5-8-1	0.9270 0.0056	0.8551 0.0056	0.7094 0.0145	Tanh	Identity
	MLP 5-8-1	0.9261 0.0057	0.8287 0.0057	0.7176 0.0149	Exponential	Exponential
	MLP 5-6-1	0.9297 0.0054	0.8689 0.0054	0.7101 0.0103	Exponential	Exponential
PEG 20000/ SNV	MLP 5-7-1	0.9912 0.0003	0.7979 0.0028	0.7501 0.0061	Exponential	Exponential
	MLP 5-4-1	0.8970 0.0036	0.8056 0.0052	0.7808 0.0064	Exponential	Identity
	MLP 5-7-1	0.8329 0.0046	0.8036 0.0043	0.7844 0.0068	Logistic	Tanh
	MLP 5-7-1	0.8154 0.0043	0.8042 0.0054	0.6151 0.0091	Logistic	Tanh
	**MLP 5-5-1**	**0.9917** **0.0002**	**0.9294** **0.00184**	**0.8533** **0.0027**	**Exponential**	**Exponential**
PEG 1500/ MSC	MLP 5-11-1	0.9998 0.0002	0.9994 0.0005	0.9985 0.0015	Exponential	Identity
	MLP 5-11-1	0.9993 0.0007	0.9988 0.0012	0.9972 0.0028	Exponential	Identity
	MLP 5-10-1	0.9966 0.0034	0.9921 0.0079	0.9920 0.0080	Exponential	Identity
	MLP 5-11-1	0.9997 0.0003	0.9996 0.0004	0.9993 0.0007	Logistic	Identity
	**MLP 5-8-1**	**0.9996** **0.0004**	**0.9994** **0.0006**	**0.9995** **0.0005**	**Logistic**	**Identity**
PEG 6000/ MSC	**MLP 5-3-1**	**0.9516** **0.0044**	**0.9311** **0.0075**	**0.8113** **0.0096**	**Tanh**	**Logistic**
	MLP 5-11-1	0.9227 0.0042	0.8667 0.0086	0.7257 0.0185	Tanh	Tanh
	MLP 5-10-1	0.7311 0.0092	0.7104 0.0128	0.6716 0.0167	Logistic	Logistic
	MLP 5-5-1	0.7693 0.0092	0.7285 0.0113	0.7182 0.0142	Tanh	Logistic
	MLP 5-4-1	0.8271 0.0087	0.7577 0.0125	0.7192 0.0128	Tanh	Logistic
PEG 20000/ MSC	MLP 5-5-1	0.9924 0.0003	0.8273 0.0031	0.6731 0.0091	Logistic	Identity
	**MLP 5-5-1**	**0.9990** **0.0003**	**0.9915** **0.005**	**0.9978** **0.0008**	**Exponential**	**Tanh**
	MLP 5-9-1	0.9868 0.0001	0.9691 0.0007	0.9528 0.0016	Exponential	Logistic
	MLP 5-5-1	0.9969 0.0004	0.7644 0.0055	0.7234 0.0084	Logistic	Logistic
	MLP 5-11-1	0.9908 0.0005	0.9392 0.0006	0.9281 0.0023	Exponential	Identity

## Data Availability

Not applicable.
